# Age-related changes in the sense of body ownership: New insights from the rubber hand illusion

**DOI:** 10.1371/journal.pone.0207528

**Published:** 2018-11-15

**Authors:** Angela Marotta, Massimiliano Zampini, Michele Tinazzi, Mirta Fiorio

**Affiliations:** 1 Department of Neuroscience, Biomedicine and Movement Sciences, University of Verona, Verona, Italy; 2 Neurology Unit, Neuroscience Department, Azienda Ospedaliera Universitaria Integrata, Verona, Italy; 3 CiMeC Center for Mind/Brain Sciences, University of Trento, Rovereto, Italy; Birkbeck University of London, UNITED KINGDOM

## Abstract

How do age-related changes affect the sense of body ownership? This study tackles this issue by means of the rubber hand illusion (RHI), a widely used experimental tool for investigating the sense of body ownership. There is ample literature on the RHI in young populations, but research on age-related changes in the RHI is still scarce. Here we extend the use of the RHI to examine the changes in the sense of body ownership related to healthy aging. Subjective reports (i.e., questionnaire) and proprioceptive drift were compared among young (n = 22, age range 20–22 years), middle-aged (n = 22, age range 44–55 years), and older adults (n = 22, age range 60–72 years). A stronger subjective experience of illusion was observed in the young and older adults as compared to the middle-aged. No differences in proprioceptive drift were found between the three groups. These findings are discussed in relation to: 1) different stages of development of perceptual and cognitive components of the sense of body ownership, and 2) compensatory mechanisms.

## Introduction

Sociologist Bryan S. Turner wrote: “*There is an obvious and prominent fact about human beings*: *They have bodies and they are bodies*” [1] (1984). This observation aptly conveys the concept of the “bodily self”, according to which our sense of the self is intimately linked to the body [2, 3]. One fundamental dimension of the bodily self is the sense of body ownership, which refers to the experience of “my body as mine” [3]. This cognitive construct relies on the multisensory integration of afferent sensory information (e.g., visual, tactile, proprioceptive, interoceptive, vestibular, and auditory stimuli) arising within the body itself [4], as well as on the coherence between this body-related information and pre-existing body representations [5].

The cognitive and neural mechanisms of the sense of body ownership have been mainly investigated in young individuals [4, 6, 7, 8], whereas less is known about this function in older adults. Ageing is an unavoidable process in living beings. In humans it is associated with changes in the physical aspect of the body, like the appearance of wrinkles, grey hair or age spots. These changes are often associated with body dissatisfaction [9], but little is known about whether they influence the bodily self. Investigating how age-related body changes interact with the sense of the self, and specifically with the sense of body ownership, can extend our knowledge about the factors that modulate the sense of body ownership and can open the way to new important lines of research for promoting wellbeing in different stages of life. The aim of this study was, therefore, to experimentally investigate the sense of body ownership in the life span by applying a well-known paradigm, the rubber hand illusion (RHI) [10] to young, middle-aged, and older participants.

In the RHI, synchronous strokes of a person’s own hidden hand and of a visible rubber hand elicit the feeling that the rubber hand belongs to the person’s own body [10]. The RHI paradigm allows to investigate both the subjective experience of feeling ownership over the rubber hand and the proprioceptive relocation of the real unseen hand toward the viewed rubber hand (i.e., proprioceptive drift). Previous studies have mapped these different aspects onto two anatomically distinct neuronal substrates, with the ventral premotor cortex processing the illusory feeling of ownership [6], whilst the inferior parietal lobule [7], right posterior insula, frontal operculum [4], and cerebellum [6] process the drift. With increasing age, brain activity in these areas changes [11, 12], probably modifying a individual’s response to the illusion.

Three recent studies tried to tackle the issue of age-related changes in the RHI [13, 14, 15]. Graham and colleague [13] used a modified version of the classical RHI paradigm (i.e., the projected RHI) in a sample aged between 20 and 60 years old. The authors found a decreased subjective feeling of ownership and a greater proprioceptive drift with increasing age. Similar results were shared by another study using the classical RHI paradigm [14], in which the subjective feeling of ownership decreased with age while proprioceptive drift remained stable. Neither study, however, systematically addressed the three main stages of adult life, i.e., youth, middle age, and older adulthood.

More recently, Palomo and collaborators [15] overcame this limit by applying the classical RHI paradigm to these three different age groups. Though they found no age-related changes in either the subjective experience of illusion or proprioceptive drift, it should be noted that the subjective measure of the illusion was derived from a composite score of items assessing different perceptual effects of the illusion. Hence, it is not clear whether potential age-related changes in the subjective component of the RHI could be unmasked by keeping the items separated. Moreover, in that study only the synchronous stroking condition was applied, although it is known that the illusion can be elicited also with asynchronous stroking [16].

In this regard there is a certain window of time in which different stimuli can be perceptually integrated even if they occur asynchronously. The time window of simultaneity influences the tolerance to asynchronies in the RHI: the wider the time window of simultaneity, the stronger the RHI in the asynchronous stimulation [16]. In the study by Palomo and colleagues [15] only synchronous stimulation was used and the age-related effects specifically related to temporal features of multisensory integration processes remained unexplored. By applying the RHI with both synchronous and asynchronous stimulation to three different age groups (young, middle-aged, and older adults), we wanted to determine whether the two subcomponents of the sense of body ownership (i.e., subjective reports and proprioceptive drift) are modified by ageing. Given the event of body changes, the progressive decline in brain structures with advancing age [17, 18, 19], and the possible differences in multisensory interactions [20], our hypothesis was that ageing should undermine the illusion. Hence, we expected to find a reduction in both subjective reports and proprioceptive drift in the older adults as compared to the young and the middle-aged adults.

## Materials and methods

### Participants

Computation of the sample size was performed with G-Power 3.1 [21]. Assuming an anticipated effect size equal to 0.2294 (derived from a medium partial eta square of 0.05 [22], an α error probability of 0.05 and a power (1-β error probability) of 0.95, the resulting total sample size is n = 54. We decided to recruit more participants to prevent any reduction in statistical power due to potential drop-outs. Sixty-six participants were recruited through advertisements at the University of Verona and were divided into three groups. Twenty-two young (13 females; mean age ± SD, 22.50 ± 2.09 years; range 20–25), 22 middle-aged (13 females; mean age 50.18 ± 3.65 years; range 44–55), and 22 older adults (10 females; mean age 64.68 ± 4.03 years; range 60–72) took part in the study. Age ranges were defined according to published literature [23, 24]. All participants were right-handed with self-reported healthy neurological history and normal or corrected-to-normal vision. All subjects gave written informed consent in accordance with the Declaration of Helsinki. The protocol was approved by the ethical committee of the University Hospital of Verona (CESC).

### Procedure

**Fig 1:**
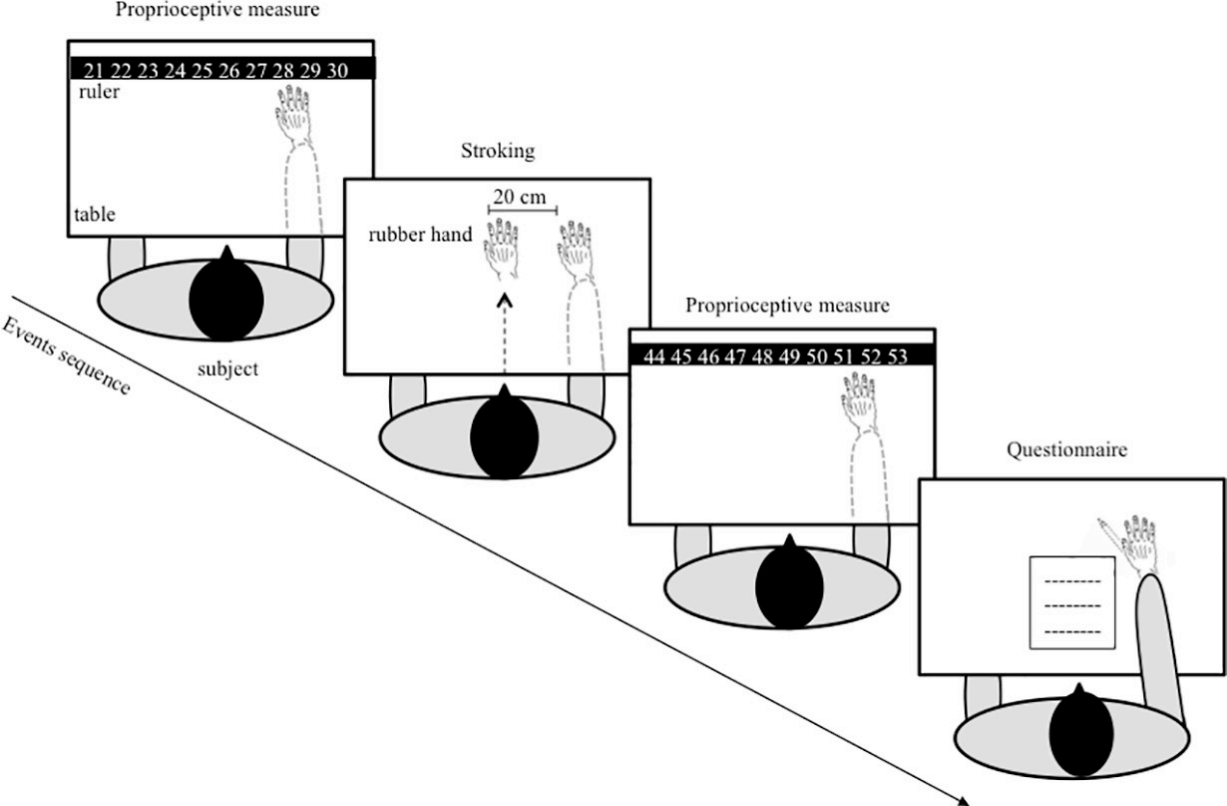
Experimental set-up and events sequence. The participant sat at a table with the forearm hidden from view and placed inside a box. Before stroking, the participant judged the felt position of her own index finger by means of a ruler. After making a proprioceptive judgment, the rubber hand was made visible and stroked synchronously or asynchronously with the participant’s hidden hand for 2 min. After stroking, the participant was again asked to judge the position of her index finger. Finally, the participant filled in the RHI questionnaire. This sequence was the same for synchronous and asynchronous stroking of the right and the left hand, thus resulting in a total of 4 conditions.

All participants underwent the RHI paradigm in a single session lasting about 1 hour. The experimental set-up consisted of two adjacent black boxes (18 x 29 cm each) placed on a table in front of the participant. A realistic artificial hand (i.e., left or right) was placed inside one box and the participant’s hand was positioned inside the other box. The distance between the index fingers of the participant’s own and the rubber hand was set at 20 cm and was constantly controlled throughout the experiment (Fig 1).

The rubber hand and the participant’s hand were stroked with two small paintbrushes either synchronously or asynchronously. During the stroking, the participants were asked to look at the rubber hand. A 2x2 experimental design was used, with two stroking modalities (synchronous, asynchronous) and two tested hands (left, right), for a total of 4 conditions with counterbalanced order across participants. To avoid fatigue and learning effects, one stroking trial of 2 minutes was performed in each condition. When the experimenter had to change the tested hand (from right to left or vice versa), the participants were asked to briefly close their eyes to avoid direct observation of the procedure until the rubber hand was placing in the box. They could then open the eyes and place their own hand into the other box. The experimenter again arranged the participant’s hand 20 cm from the rubber hand.

The subjective experience of the illusion was measured by means of a 9-statement questionnaire [10]. Based on the feelings elicited during the stroking phase, the participants rated their agreement or disagreement with each statement on a rating scale from 0 (“I totally disagree”) to 10 (“I totally agree”). The first three statements (experimental statements) usually receive higher scores after synchronous stroking and are therefore considered as being the most sensitive to capture the illusory embodiment of the rubber hand (i.e., incorporating the rubber hand in pre-existing representations of the body) [25]. More precisely, each statement describes different perceptual effects related to the embodiment of the rubber hand. Statement 1 (S1: “It seemed as if I felt the paintbrushes touching my finger where I saw the rubber hand being touched”) refers to an illusory localization of the touch over the artificial hand; Statement 2 (S2: “It seemed like the touch I felt was caused by the paintbrushes touching the rubber hand”), refers to a causal link between vision and touch; Statement 3 (S3: “I felt as if the rubber hand was my own hand”) refers to the illusory feeling of ownership over the rubber hand.

The other six statements (control statements) describe other perceptual effects that are less or not related to the illusory embodiment of the rubber hand. These statements serve as control for compliance, since they usually receive lower agreement scores than the experimental statements.

The illusion was also measured by means of proprioceptive drift, which can be defined as a recalibration of the perceived position of the participant’s own hand toward the rubber hand. To obtain this measure, before and after each stroking phase the participants were asked to verbally indicate the perceived position of their index finger with respect to a ruler placed in front of them. The starting position of the ruler was changed trial by trial in order to avoid response bias. During this procedure, the real and the artificial hands were hidden from the participant’s view by closing the boxes. Proprioceptive drift was computed by subtracting the estimation of finger positions before and after the stroking phase. Proprioceptive drift usually occurs after synchronous but not asynchronous stroking [5, 10].

### Data analyses

Age and gender distributions were analyzed by means of independent samples t-test and chi-squared, respectively.

Data were first checked for normality using the Shapiro-Wilk test (p>0.05). Since the data were not normally distributed, statistical analyses were performed using nonparametric tests. Data are represented as box plots displaying the median values. Outlier values (represented in the box plots) were included in the analysis.

Preliminary analyses were carried out to verify whether subjective scores for the questionnaire statements and proprioceptive drift were affected by hand laterality in the overall sample (n = 66). To this aim, the Wilcoxon signed rank test was used for comparisons between the left and the right hand.

The subjective scores for questionnaire items and proprioceptive drift were then further analyzed in two steps. First, the RHI was analyzed within each group. The synchronous and asynchronous stroking conditions within each group were compared using the Wilcoxon signed rank test. Second, the RHI was analyzed between groups. The between-group differences in the synchronous and asynchronous stroking conditions were analyzed by means of the Kruskal-Wallis test. Post-hoc comparisons were performed using the Mann-Whitney U-Test. P values <0.05 were considered statistically significant. Bonferroni correction was applied where necessary.

## Results

The independent samples t-test confirmed that the three groups differed for age (for all comparisons, p<0.001). No differences were found for gender distribution (all, p>0.371). The illusion was similar for the right and the left hand: no differences in the subjective scores for the questionnaire items (for all comparisons, p>0.151) except for S2 in the asynchronous stroking condition (p = 0.043). Moreover, no differences between hands were found for proprioceptive drift (for all comparisons, p>0.055). Accordingly, the hypothesis of a potential effect of laterality in the amount of illusion (as assessed by both the questionnaire and proprioceptive drift) in our sample was rejected. The subjective scores and proprioceptive drift obtained from the left and right hands were averaged together and entered in the main analyses.

### The rubber hand illusion within each group

**Fig 2:**
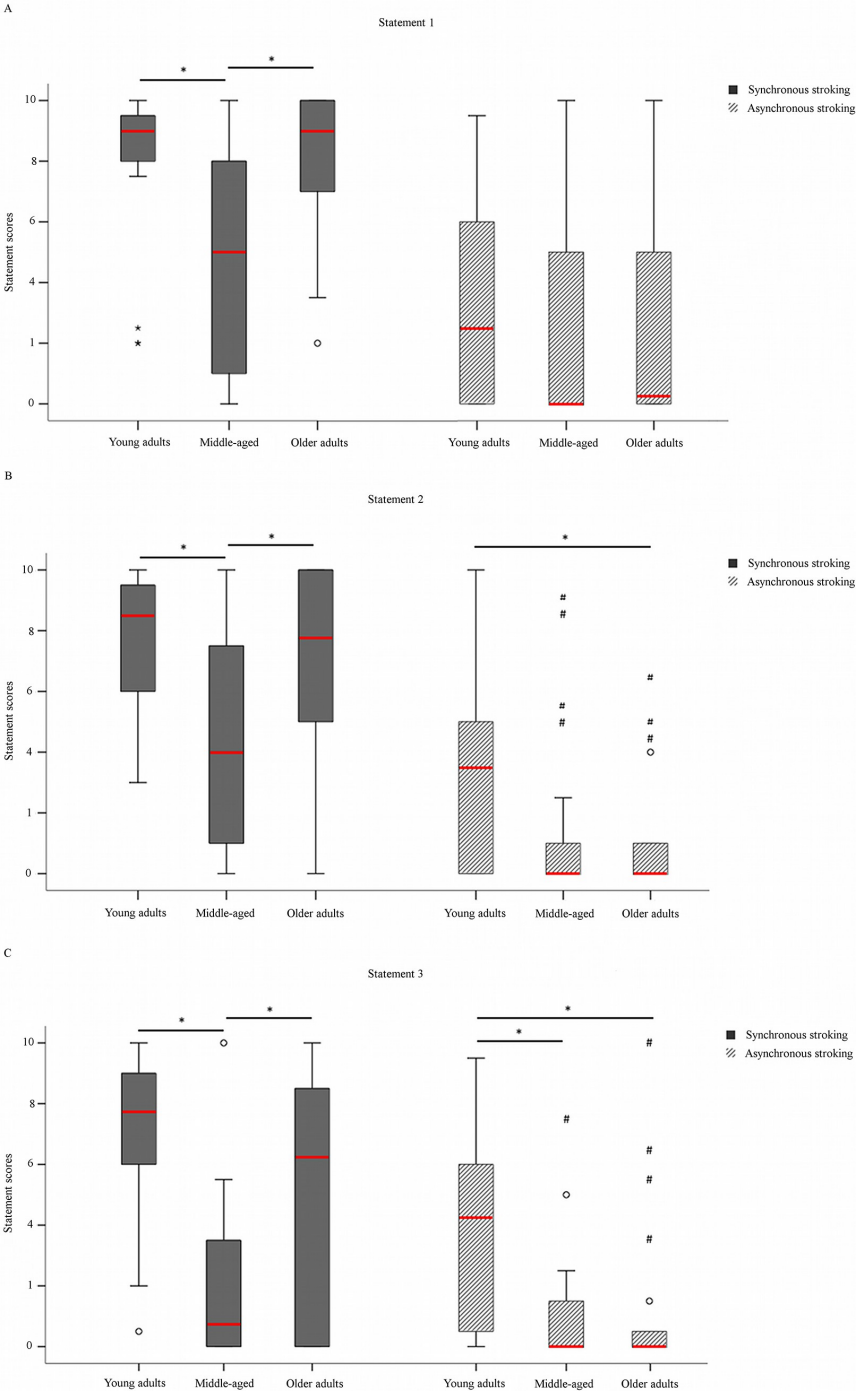
Boxplot of the scores for the experimental statements in the young, middle-aged, and older adult groups. In the synchronous stroking condition (gray columns), scores for all experimental statements were higher in the young and the older adult groups than in the middle-aged group. This pattern qualitatively describes a characteristic U-shape of the illusory embodiment of the rubber hand across different stages of life. Between-group differences were found also for the asynchronous stroking condition (gray-striped columns) at statements 2 and 3. The scores for these statements were higher in the young adult group than in the other two groups. *Significant values (p<0.05); °outliers (e.g., values falling between 1.5 and 3 times above or below the interquartile range); # extreme outliers (e.g., values more than three times above or below the interquartile range). Red bars represent median values.

In the young adults group, the subjective scores for the questionnaire were generally higher after synchronous than asynchronous stroking, confirming the typical pattern observed in the RHI paradigm. The effect of stroking was significant for the experimental statements strictly related to the illusion (S1: Z = -4.077, p < 0.001, r = 0.869; S2: Z = -3.849, p<0.001, r = 0.821; S3: Z = -3.657, p<0.001, r = 0.780) (Fig 2). A significant effect of stroking was observed also for all control statements, except for S8 (S4: Z = -2.553, p = 0.011, r = 0.544; S5: Z = -2.139, p = 0.032, r = 0.456; S6: Z = -3.280, p = 0.001, r = 0.699; S7: Z = -2.445, p = 0.014, r = 0.521; S9: Z = -2.717, p = 0.007, r = 0.579).

**Fig 3:**
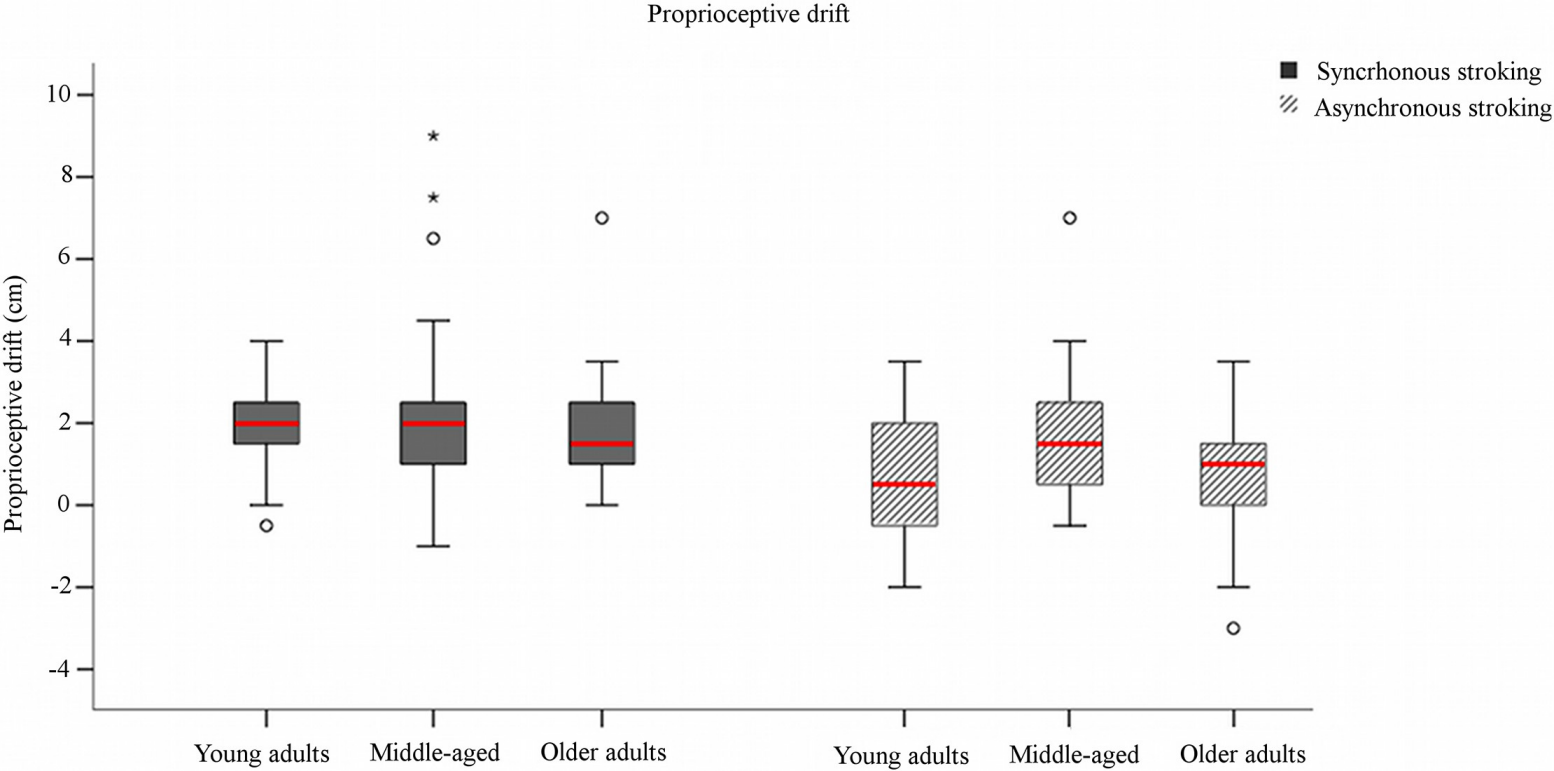
Boxplot of proprioceptive drift in the young adults, middle-aged, and older adults groups. Proprioceptive drift was similar between groups in both the synchronous (gray columns) and the asynchronous (gray-striped columns) stroking conditions, suggesting that age does not affect the illusory displacement of the real hand towards the rubber hand. °Outliers (e.g., values falling between 1.5 and 3 times above or below the interquartile range); # extreme outliers (e.g., values more than three times above or below the interquartile range). Red bars represent median values.

Also, proprioceptive drift was significantly higher after synchronous than asynchronous stroking (Z = -2.964, p = 0.003, r = 0.632) (Fig 3). Again, this result was in line with the typical pattern observed in the RHI and confirmed the paradigm’s validity.

In the middle-aged group, the subjective scores for S1 and S2 were higher after synchronous than after asynchronous stroking (S1: Z = -2.701, p = 0.007, r = 0.576; S2: Z = -2.937, p = 0.003, r = 0.626). Conversely, no difference between stroking conditions was found for S3 (Z = -1.226, p = 0.220), which specifically assesses the feeling of ownership over the rubber hand. This new finding suggests that the subjective component of the illusion in the middle-aged group does not follow the typical pattern. Significant differences between conditions were found for control statements S4 and S7, which were rated higher after synchronous than after asynchronous stroking (S4: Z = -2.665, p = 0.004, r = 0.568; S7: Z = -2.334, p = 0.020, r = 0.498). Also proprioceptive drift was outside the typical pattern, as revealed by the lack of a significant difference between stroking conditions (Z = -0.958, p = 0.338) (Fig 3). This suggests that synchronous stroking in the middle-aged group did not induce a stronger recalibration of the own hand than asynchronous stroking.

The older adults showed a pattern resembling that of the young adults: the experimental statements were rated higher after synchronous than after asynchronous stroking (S1: Z = -4.019, p<0.001, r = 0.857; S2: Z = -3.839, p<0.001, r = 0.840; S3: Z = -3.417, p = 0.001, r = 0.729) (Fig 2). Moreover, higher scores were noted for some control statements after synchronous stroking (S6: Z = -2.313, p = 0.021, r = 0.493; S7: Z = -2.493, p = 0.013, r = 0.532; S9: Z = -2.351, p = 0.019, r = 0.501). Finally, as seen in the young adults group, the type of stroking influenced proprioceptive drift, as demonstrated by the greater drift after synchronous than after asynchronous stroking (Z = -2.302, p = 0.021, r = 0.491) (Fig 3).

### The rubber hand illusion between groups

The Kruskal-Wallis test revealed significant between-group differences in the scores for the three experimental statements in the synchronous stroking condition (S1: χ^2^(2) = 14.098, p = 0.001; S2: χ^2^(2) = 13.136, p = 0.001; S3: χ^2^(2) = 20.380, p<0.001). Post-hoc comparisons (critical p≤0.016 after Bonferroni correction) revealed that this difference was due to higher scores in the young and the older adult groups versus the middle-aged group (young adults vs. middle-aged: S1: Z = -3.277, p = 0.001, effect size = 0.250; S2: Z = -3.430, p = 0.001, effect size = 0.267; S3: Z = -4.624, p<0.001, effect size = 0.486; older adults vs. middle-aged: S1: Z = -3.182, p = 0.001, effect size = 0.230; S2: Z = -2.777, p = 0.005, effect size = 0.175; S3: Z = -2.516, p = 0.012, effect size = 0.144) (Fig 2A, 2B and 2C). These findings suggest that the subjective components of the illusion were reduced in the middle-aged group as compared to the other two groups. Between-group differences emerged also after asynchronous stroking at S2 (χ^2^(2) = 13.136, p = 0.013) and S3 (χ^2^(2) = 14.751, p = 0.001). Post-hoc comparisons revealed that in the asynchronous condition the young adults gave higher scores to S2 than the older adults (Z = -2.551, p = 0.011, effect size = 0.148) and that they gave higher scores to S3 than either the middle-aged (Z = -3.369, p = 0.002, effect size = 0.258) or the older adults (Z = -3.257, p = 0.001, effect size = 0.241) (Fig 2B and 2C).

Significant between-group differences were also observed for S7 in both stroking conditions (synchronous: χ^2^(2) = 9.010, p = 0.011; asynchronous: χ^2^(2) = 12.476, p = 0.002). Post-hoc comparisons showed that the young adults gave higher scores than the middle-aged group after synchronous stroking (Z = -3.254, p< 0.001, effect size = 0.241), whereas after asynchronous stroking they gave higher scores than either the middle-aged (Z = -2.866, p = 0.004, effect size = 0.187) or the older adults (p = 0.003). Finally, no between-group difference was found for proprioceptive drift (synchronous: Z = 0.506, p = 0.777; asynchronous: Z = 4.649, p = 0.098) (Fig 3).

## Discussion

This study investigated age-related changes in the sense of body ownership by means of the RHI. Though there is copious literature about the sense of body ownership in young populations, little is known about the changes that this function undergoes in other stages of life (e.g., middle and older age). By applying the RHI to participants from three age groups (young, middle-aged, and older adults), we discovered that the subjective component of body ownership seems to follow a U-shape. These findings could suggest for the first time that the mechanisms underlying the illusory embodiment of the rubber hand (supposedly multisensory integration and flexibility of body representations) may change across the human life span, with higher illusion noted in young and older adults as compared to the middle-aged.

### Subjective experience of body ownership traces a U-shape across the life span

Questionnaire data showed high variability, indicating individual differences in susceptibility to the illusion [26, 27, 28]. Despite this variability, a consistent pattern could be discerned. The experience of the illusion as measured with the questionnaire differed between the three groups. Both the young and the older adults rated all three illusion-related statements (S1, S2, and S3) higher after synchronous than after asynchronous stroking. The middle-aged group presented with a slightly different pattern of responses, with higher scores given for S1 and S2 but not for S3 after synchronous as compared to asynchronous stroking. These findings go against our initial hypothesis of a progressive reduction of the RHI with age. Of note, S1, S2, and S3 refer to different dimensions of embodiment [29]: while S1 (“It seemed as if I felt the paintbrushes touching my finger where I saw the rubber hand being touched”) and S2 (“It seemed like the touch I felt was caused by the paintbrushes touching the rubber hand”) are related to the localization of touch, S3 (“I felt as if the rubber hand was my own hand”) is more specifically related to the feeling of ownership over the rubber hand [29]. Localizing touch and feeling ownership involve different underlying mechanisms [30]. While localizing touch over the rubber hand (S1 and S2) mainly relies on the temporal congruency between visual and tactile stimuli, feeling ownership (S3) requires additional processing to verify whether the incoming body-related information is coherent with the inner representations of body appearance (the body image) [5, 31]. Our findings suggest that multisensory integration of conflicting visuo-tactile information was still possible to some extent in the middle-aged group, whereas the integration of an external object into the body representation did not fully occur. Conversely, the young and the older adults experienced both the localization of touch (S1 and S2) and the feeling of ownership (S3) after synchronous stroking. These findings hint at an effective multisensory integration of conflicting visual, tactile, and proprioceptive information and at a flexible representation of the body, which allows for full incorporation of the rubber hand into the internal body model, thus leading to the feeling of ownership.

Other studies have found similar behavioral performance between young and older adults and have explained it in terms of compensatory brain activity occurring with age [11]. For instance, a previous study demonstrated that although behavioral performance on a motor task was similar for young and older adults (i.e., sequential finger movements), the older adults presented with different activations in specific brain regions [11]. As compared to the young adults, activation in the bilateral premotor cortices and the anterior parietal area was higher in the older adults in which additional recruitment of the posterior parietal cortex was also noted [11]. Interestingly, these brain areas are involved in different components of the RHI: the premotor cortex (especially in its ventral part) is associated with the subjective feeling of ownership [6, 32], whereas the posterior parietal cortex seems to be involved in proprioceptive drift [7]. Both areas have important roles also in the multisensory integration of visual and tactile stimuli [33, 34]. Moreover, the anterior parietal area is involved in the comparison between the current state of the body and the postural and anatomical features of the body part to be incorporated [4, 35]. Although different tasks were used in our and in the previous study [11], we could speculate that the older adults performed similarly to the young adults by virtue of compensatory mechanisms in brain regions involved in the RHI. This assumption, however, needs to be investigated in future studies.

On comparing the three groups, we found that the young and the older adults rated S1, S2, and S3 higher than the middle-aged group after synchronous stroking, indicating that they experienced the illusory localization of touch and the feeling of ownership over the rubber hand *more vividly* than the middle-aged group. The lack of differences between the young and the older adults suggests that the two groups experienced the illusion very similarly. These findings contrast with a recent RHI study that reported no age-related differences in the subjective experience of ownership [15]. This discrepancy might be explained by methodological differences. Palomo and collaborators [15] computed the body ownership score as the average of five different items and the localization of touch as the average of two different items. Hence, merging different items into single composite scores could have masked age-related differences in specific perceptual effects related to the illusion. Differently, we analyzed between-group differences by separately focusing on the perceptual effects of the illusion. This allowed us to show for the first time that very specific subjective components of the sense of body ownership change across different ages. Qualitatively, these changes form a U-shape, with a reduced RHI for the middle-aged compared to the other groups and a similar pattern shared by the young and the older adults. This peculiar pattern of responses could be interpreted as a behavioral consequence of the development of perceptual and cognitive functions related to the sense of body ownership.

With regard to perceptual functions, multisensory integration is crucial in the RHI, as the paradigm involves the ability to integrate visual input with tactile and proprioceptive input. And while the multisensory information in the RHI is correlated in the temporal domain (i.e., the illusion occurs when visual and tactile inputs are synchronous), the same sensory inputs are actually separated in the spatial domain (i.e., the visual input comes from the rubber hand, whereas the tactile and proprioceptive input comes from the person’s own hand). Typically, both temporal and spatial contingencies between stimuli determine a higher probability that multisensory inputs are bound together [36, 37]. Hence, the stronger RHI found in the young and the older adults suggests that, despite being spatially separated, the visual and tactile inputs were more successfully bound together in both these age groups.

Although multisensory integration is the main cause of the illusion [3], it is not sufficient *per se* to evoke the illusory embodiment of the rubber hand. Perceiving the rubber hand as belonging to one’s own body requires additional top-down cognitive processing to verify whether the multisensory information is coherent with pre-existing representations of the body [5]. The feeling of ownership over the rubber hand arises only if the multisensory percept derived from synchronous stroking of the participant’s hand and the rubber hand is admitted to be part of the body representation. This requires some degree of plasticity of body representation [31], which is reflected in the individual’s response to the RHI [38].

The lower illusory embodiment of the rubber hand in the middle-aged group suggests a reduced flexibility of body representation as compared to the other two groups. Since both young and older adults are at a time in life when they face greater changes in physical appearance than during middle age, they may well require more flexibility of body representation to maintain a coherent sense of self [39]. Several RHI studies found a strong illusion in young adults [4, 29, 30], indicating high malleability of body representation at this stage of life. Conversely, future studies are needed to examine the flexibility of body representation in older adults.

### Stability of proprioceptive drift across ages

The young and the older adults gave similar responses for proprioceptive drift, with higher drift noted after synchronous than asynchronous stroking. In both groups, synchronous stroking also evoked a shift in the perceived position of the subject’s own hand toward the rubber hand.

Conversely, no differences in proprioceptive drift after synchronous and asynchronous stroking were noted for the middle-aged group in which we observed a dissociation between subjective reports (i.e., Statements 1 and 2) and proprioceptive drift. Indeed, while synchronous stroking was able to elicit a subjective experience of localizing touch over the artificial hand, it did not successfully determine a sensitive increase in the displacement of the subject’s own hand toward the rubber hand. This observation is in line with previous studies that found a dissociation between proprioceptive drift and subjective reports of illusion [40, 41, 42, 43]. Since the subjective experience of localizing touch over the rubber hand relies mainly on visuo-tactile integration, whereas proprioceptive drift relies on visuo-proprioceptive information, these findings might suggest that the middle-aged participants were more resistant to integrating visual and proprioceptive information conflicting with visuo-tactile information.

We noted a similar dissociation between subjective reports and proprioceptive drift also in the between-group comparisons. Although we did find significant between-group differences in the subjective reports (i.e., questionnaire), no group effect was seen for proprioceptive drift after either synchronous or asynchronous stroking. Again, this finding contrasts with our initial prediction of a progressive reduction of the illusion with age, suggesting that proprioceptive drift remains stable from young to older adulthood. Interestingly, a previous study demonstrated that by 10 years of age the proprioceptive drift reaches the adult level [44]. Our study extends these findings by suggesting that proprioceptive drift remains stable also in middle-age and older adulthood.

Finding contrasting results between the questionnaire ratings and the proprioceptive drift is not new. Indeed, subjective reports of the illusion and proprioceptive drift have often been found to dissociate. Evidence for this dissociation comes from previous studies demonstrating a shift in the perceived position of the participant’s hand toward the rubber hand even in the absence of a subjectively reported feeling of ownership, and vice versa [40, 41, 42, 43]. These findings suggest that the proprioceptive drift and the subjective feeling of ownership are different, independent processes. Moreover, a study investigating the relationship between hand position sense and the rubber hand illusion, demonstrated that the proprioceptive drift might be strongly affected by the manipulation of hand position without influencing the subjective feeling of ownership as measured by the questionnaire [40]. These evidence rise against a causal role of the proprioceptive drift in the illusory feeling of ownership [40], thus questioning its use as measure of ownership in the rubber hand illusion [40, 41, 42, 43].

### Other perceptual effects evoked by the RHI paradigm

The higher scores for S2 and S3 noted also after asynchronous stroking in the young adults suggest that the asynchronous presentation of visual and tactile stimuli evoked a stronger illusion than in the other two groups. A recent study showed that the RHI can be evoked even in the asynchronous condition and that the degree of asynchrony tolerated in the RHI depends on the individual window of time in which multisensory integration can occur [16]. More precisely, individuals with a larger temporal binding window showed stronger illusion after asynchronous stimulation [16]. Our findings suggest that the temporal binding window was larger in the young adults, allowing them to better tolerate asynchrony in the RHI.

The control statements also received some degree of attention. Although the median scores were very low (for all groups and conditions, median values <5), some were higher after synchronous than after asynchronous stroking for the young (i.e., S4, S6, S7, and S9), the middle-aged (i.e., S7), and the older adults (i.e., S6, S7, and S9). These results suggest that all participants were sensitive to other perceptual effects related to synchronous stimulation, such as feeling their own hand shifting towards the rubber hand (S4) and perceiving similarity between their own and the rubber hand (S7, S9). Between-group differences were noted for S7, with higher scores observed in the young adults after synchronous and asynchronous stroking as compared to the middle-aged and the older adults. In fact, Statement 7 refers to a sensation (“It felt as if my hand were turning rubbery”) that has been associated with embodiment of the rubber hand [29]. The illusory effects were more pervasive in the young adults than in either the older or the middle-aged adults.

## Conclusions

Ageing is an unavoidable process in living beings. Age-related changes are often associated with body dissatisfaction [9]. As they can influence socialization, understanding how age-related body changes interact with the sense of the self is crucial for promoting wellbeing in different stages of life. Little is known about the mechanisms underlying the relationship between ageing and the self. Our study sheds new light on this topic by focusing on the age-dependent changes in the sense of body ownership. Like the young adults, the older adults presented with higher flexibility of body representation as compared to the middle-aged. Moreover, the physiological decline of the sensory system notwithstanding, the older adults were noted to preserve the ability to integrate bodily-related information in a coherent percept. By contrast, the middle-aged adults were more resistant to the body ownership illusion, suggesting a higher stability of body representation compared to both the young and the older adults. Since we used the RHI paradigm, these findings are limited to the embodiment of one body part (e.g., the hand), which is not as salient in the evaluation of body appearance as the whole body [45]. A future area of focus would be to address age-related changes in other types of illusion, such as the full-body illusion [46], which may be more closely related to body appearance.

## References

[1] Turner B. The body and society: exploration in social theory NY: Basil Blackwell; 1984.

[2] Azañón E, Tamè L, Maravita A, Linkenauger SA, Ferrè ER, Tajadura-Jiménez A, et al Multimodal contributions to body representation. Multisensory research. 2016; 29:635–661. 10.1163/22134808-00002531

[3] Tsakiris M. The multisensory basis of the self: from body to identity to others. Q J Exp Psychol. 2017; 70: 597–609. 10.1080/17470218.2016.1181768 27100132 10.1080/17470218.2016.1181768 PMC5214748

[4] Tsakiris M, Hesse M, Boy C, Haggard P, Fink GR. Neural correlates of body-ownership: A sensory network for bodily self-consciousness. Cereb Cortex. 2007; 17: 2235–2244. 10.1093/cercor/bhl131 17138596 10.1093/cercor/bhl131

[5] Costantini M, Haggard P. The rubber hand illusion: sensitivity and reference frame for body ownership. Conscious and Cogn. 2007; 16: 229–240. 10.1016/j.concog.2007.01.001 17317221 10.1016/j.concog.2007.01.001

[6] Ehrsson HH, Spence C, Passingham RE. That’s my hand! Activity in premotor cortex reflects feeling of ownership of a limb. Science. 2004; 305: 875–877. 10.1126/science.1097011 15232072 10.1126/science.1097011

[7] Kammers MP, Verhagen L, Dijkerman HC, Hogendoorn H, de Vignemont F, Schutter DJ. Is this hand for real? Attenuation of the rubber hand illusion by transcranial magnetic stimulation over the inferior parietal lobule. J Cogn Neurosci. 2009; 21; 1311–1320. 10.1162/jocn.2009.21095 18752397 10.1162/jocn.2009.21095

[8] Moseley GL, Olthof N, Venema A, Don S, Wijers M, Gallace A, et al Psychologically induced cooling of a specific body part caused by the illusory ownership of an artificial counterpart. Proc Natl Acad Sci USA. 2008; 105: 13169–13173. 10.1073/pnas.0803768105 18725630 10.1073/pnas.0803768105 PMC2529116

[9] Clarke LH, Korotchenko A. Aging and the body: a review. Can J Aging. 2011; 30:495–510. 10.1017/S0714980811000274 24976674 10.1017/S0714980811000274 PMC4072651

[10] Botvinick M, Cohen J. Rubber hands “feel” touch that eyes see. Nature. 1998; 391:756 10.1038/35784 9486643 10.1038/35784

[11] Wu T, Hallett M. The influence of normal human ageing on automatic movements. J Physiol. 2005; 562: 605–615. 10.1113/jphysiol.2004.076042 15513939 10.1113/jphysiol.2004.076042 PMC1665504

[12] Bernard JA, Seidler RD. Moving Forward: Age Effects on the Cerebellum Underlie Cognitive and Motor Declines. Neurosci Biobehav Rev. 2014; 0: 193–207. 10.1016/j.neubiorev.2014.02.011 10.1016/j.neubiorev.2014.02.011 PMC4024443 24594194

[13] Graham KT, Martin-Iverson MT, Holmes NP, Waters FA. The projected hand illusion: component structure in a community sample and association with demographics, cognition, and psychotic-like experiences. Atten Percept Psycho. 2015; 77: 2017–2019. 10.3758/s13414-014-0748-6 25120179 10.3758/s13414-014-0748-6

[14] Kállai J, Kinces P, Lábadi B, Dorn K, Szlocsányi T, Darnai G, et al Multisensory integration and age-dependent sensitivity to body representation modification induced by the rubber hand illusion. Cogn Process- 2017: 18; 349–357. 10.1007/s10339-017-0827-4 28780698 10.1007/s10339-017-0827-4

[15] Palomo P, Borrego A, Cebolla A, Llorens R, Demarzo M, Baños RM. Subjective, behavioral, and physiological responses to the rubber hand illusion do not vary with age in the adult phase. Conscious Cogn. 2017; 58: 90–96. 10.1016/j.concog.2017.10.014 29103810 10.1016/j.concog.2017.10.014

[16] Costantini M, Robinson J, Migliorati D, Donno B, Ferri F, Northoff G. Temporal limits on rubber hand illusion reflect individuals’ temporal resolution in multisensory perception. Cognition. 2016; 157: 39–48. 10.1016/j.cognition.2016.08.010 27592410 10.1016/j.cognition.2016.08.010

[17] Glisky EL. Changes in cognitive function in human aging In Brain Aging: Models, Methods, and Mechanisms. Boca Raton (FL): CRC Press; 2007.

[18] Grady GL, Springer MV, Hongwanishkul D, McIntosh AR, Winocour G. Age-related changes in brain activity across the adult lifespan. J Cogn Neurosci. 2006; 18: 227–241. 10.1162/089892906775783705 16494683 10.1162/089892906775783705

[19] Hedman AM, van Haren NEM, Schnack HG, Kahn RS, Hulshoff Pol HE. Human brain changes across the life span: a review of 56 longitudinal magnetic resonance imaging studies. Hum Brain Mapp. 2012; 33: 1987–2002. 10.1002/hbm.21334 21915942 10.1002/hbm.21334 PMC6870052

[20] de Dieuleveult AL, Siemonsma PC, van Erp JB, Brouwer AM. Effects of ageing in multisensory perception: a systematic review. Fron Aging Neurosci. 2017; 9: 80 10.3389/fnagi.2017.00080 28400727 10.3389/fnagi.2017.00080 PMC5368230

[21] Faul F, Erdfelder E, Lang AG, Buchner A. G*Power 3: a flexible statistical power analysis program for the social, behavioral, and biomedical sciences. Behav Res Methods. 2007;39(2):175–91. 17695343 10.3758/bf03193146

[22] Bakeman R. Recommended effect size statistics for repeated measures designs. Behavior Research Methods 2005; 37: 379–384. 16405133 10.3758/bf03192707

[23] Yang L, Hasher L. The enhanced effects of pictorial distraction in older adults. J Gerontol B Psychol Sci Soc Sci. 2007; 62: 230–233. 10.1093/geronb/62.4.P230 10.1093/geronb/62.4.p230 17673533

[24] Ziebland S, Robertson J, Jay J, Neil A. Body image and weight change in middle age: a qualitative study. Int J Obes. 2002; 26:1083–1091. 10.1038/sj.ijo.0802049 12119574 10.1038/sj.ijo.0802049

[25] Walsh E, Guilmette DN, Longo M, Moore JW, Oakley DA, Halligan PW, et al Are you suggesting that’s my hand? The relation between hypnotic suggestibility and the rubber hand illusion. Perception. 2015; 44: 709–723. 10.1177/0301006615594266 26489211 10.1177/0301006615594266

[26] Marotta A, Tinazzi M, Cavedini C, Zampini M, Fiorio M. Individual Differences in the Rubber Hand Illusion Are Related to Sensory Suggestibility. Plos One 2016; 11: e0168489 10.1371/journal.pone.0168489 27977783 10.1371/journal.pone.0168489 PMC5158054

[27] Kállai J, Hegedüs G, Feldmann Á, Rózsa S, Darnai G, Herold R, et al Temperament and psychopathological syndromes specific susceptibility for rubber hand illusion. Psychiatry Res. 2015; 229: 410–9. 10.1016/j.psychres.2015.05.109 26160198 10.1016/j.psychres.2015.05.109

[28] Haans A, Kaiser FG, Bouwhuis DG, Ijsselsteijn WA. Individual differences in the rubber-hand illusion: predicting self-reports of people’s personal experiences. Acta Psychol (Amst). 2012; 141:169–77. 22964058 10.1016/j.actpsy.2012.07.016

[29] Longo M, Schüür F, Kammers MPM, Tsakiris M, Haggard P. What is embodiment? A psychometric approach. Cognition. 2008; 107: 978–998. 10.1016/j.cognition.2007.12.004 18262508 10.1016/j.cognition.2007.12.004

[30] Tsakiris M, Haggard P. The rubber hand illusion revisited: visuotactile integration and self-attribution. J Exp Psychol Hum Percept Perform. 2005; 31: 80–91. 10.1037/0096-1523.31.1.80 15709864 10.1037/0096-1523.31.1.80

[31] Longo M, Haggard P. What is like to have a body? Curr Dir Psychol Sci. 2012; 21: 140–145. 10.1177/0963721411434982

[32] Zeller D, Gross C, Bartsch A, Johansen-Berg H, Classen J. Ventral premotor cortex may be required for dynamic changes in the feeling of limb ownership: a lesion study. J Neurosci. 2011; 31: 4852–4857. 10.1523/JNEUROSCI.5154-10.2011 21451023 10.1523/JNEUROSCI.5154-10.2011 PMC3119817

[33] Gentile G, Petkova V, Ehrsson E. Integration of visual and tactile signals from the hand in the human brain: An fMRI study. J Neurophysiol. 2011; 105: 910–922. 10.1152/jn.00840.2010 21148091 10.1152/jn.00840.2010 PMC3059180

[34] Pasalar S, Ro T, Beuchamp M.S. TMS of posterior parietal cortex disrupts visual tactile multisensory integration. Eur J Neurosci. 2010; 31: 1783–90. 10.1111/j.1460-9568.2010.07193.x 20584182 10.1111/j.1460-9568.2010.07193.x PMC2994715

[35] Press C, Heyes C, Haggard P, Eimer M. (2008). Visuotactile Learning and body representation: an ERP study with rubber hands and rubber objects. J Cogn Neurosci, 20, 312–323. 10.1162/jocn.2008.20022 18275337 10.1162/jocn.2008.20022 PMC2373573

[36] Kersten D, Yuille AL. Bayesian models of object perception. Curr Opin Neurobiol. 2003; 13; 1–9. 10.1016/S0959-4388(03)00042-4 10.1016/s0959-4388(03)00042-4 12744967

[37] Mamassian P, Landy M, Maloney LT. Bayesian modeling of visual perception In Probabilistic models of the brain. Cambridge, MA: MIT Press; 2002.

[38] Tsakiris M, Carpenter L, James D, Fotopoulou A. Hands only illusion: multisensory integration elicits sense of ownership for body parts but not for non-corporeal objects. Exp Brain Res. 2010; 204: 343–352. 10.1007/s00221-009-2039-3 19820918 10.1007/s00221-009-2039-3

[39] Tajadura-Jiménez A, Longo M, Coleman R, Tsakiris M. The person in the mirror: using the enfacement illusion to investigate the experiential structure of self-identification. Conscious Cogn. 2012; 21: 1725–1738. 10.1016/j.concog.2012.10.004 23123685 10.1016/j.concog.2012.10.004 PMC3759963

[40] Abdulkarim Z, Ehrsson HH. No causal link between changes in hand position sense and feeling of limb ownership in the rubber hand illusion. Atten Percept Psychophys. 2010; 78:707–720. 10.3758/s13414-015-1016-0 26555651 10.3758/s13414-015-1016-0 PMC4744264

[41] Holle H, McLatchie N, Maurer S, Ward J. Proprioceptive drift without illusion of ownership for rotated hands in the “rubber hand illusion” paradigm. Cogn Neurosci. 2011; 2: 171–178. 10.1080/17588928.2011.603828 24168532 10.1080/17588928.2011.603828

[42] Pavani F, Zampini M. The role of hand size in the fake-hand illusion paradigm. Perception. 2007; 36: 1547–1554. 10.1068/p5853 18265837 10.1068/p5853

[43] Rohde M, Di Luca M, Ernst MO. The Rubber Hand Illusion: feeling of ownership and proprioceptive drift do not go hand in hand. Plos One. 2011; 6:e21659 10.1371/journal.pone.0021659 21738756 10.1371/journal.pone.0021659 PMC3125296

[44] Cowie D, Sterling S, Bremner A. “The development of multisensory body representation and awareness continues to10 years of age: Evidence from the rubber hand illusion. J Exp Child Psychol. 2016; 142:230–8. 10.1016/j.jecp.2015.10.003 26601752 10.1016/j.jecp.2015.10.003

[45] Mussap AJ, Salton N. A rubber hand illusion reveals a relationship between perceptual body image and unhealthy body change. J Health Psychol. 2006; 11: 627–639. 10.1177/1359105306065022 16769741 10.1177/1359105306065022

[46] Ehrsson HH. The experimental induction of out-of-body experiences. Science. 2007; 317: 1048 10.1126/science.1142175 17717177 10.1126/science.1142175

